# A Fast and Automated Strategy for the Identification and Risk Assessment of Unknown Substances (IAS/NIAS) in Plastic Food Contact Materials by GC-Q-Orbitrap HRMS: Recycled LDPE as a Proof-of-Concept

**DOI:** 10.3390/toxics9110283

**Published:** 2021-11-01

**Authors:** Pablo Miralles, Vicent Yusà, Adriana Pineda, Clara Coscollà

**Affiliations:** 1 Foundation for the Promotion of Health and Biomedical Research in the Valencian Region (FISABIO-Public Health), Avinguda Catalunya 21, 46020 Valencia, Spain; miralles_pabiba@gva.es (P.M.); yusa_vic@gva.es (V.Y.); 2 Public Health Laboratory of Valencia, Avinguda Catalunya 21, 46020 Valencia, Spain; 3 Cadel Deinking S.L., Calle Artesanos 4, 03690 Sant Vicent del Raspeig, Alicante, Spain; adriana.pineda@cadeldeinking.com

**Keywords:** food contact materials, gas chromatography, high-resolution mass spectrometry, low-density polyethylene, non-intentionally added substances, untargeted analysis

## Abstract

A fast and automated approach has been developed for the tentative identification and risk assessment of unknown substances in plastic food contact materials (FCM) by GC-Q-Orbitrap HRMS. The proposed approach combines GC-HRMS full scan data acquisition coupled to Compound Discoverer™ 3.2 software for automated data processing and compound identification. To perform the tentative identification of the detected features, a restrictive set of identification criteria was used, including matching with the NIST Mass Spectral Library, exact mass of annotated fragments, and retention index calculation. After the tentative identification, a risk assessment of the identified substances was performed by using the threshold of toxicological concern (TTC) approach. This strategy has been applied to recycled low-density polyethylene (LDPE), which could be used as FCM, as a proof-of-concept demonstration. In the analyzed sample, 374 features were detected, of which 83 were tentatively identified after examination of the identification criteria. Most of these were additives, such as plasticizers, used in a wide variety of plastic applications, oligomers of LDPE, and substances with chemical, industrial, or cosmetic applications. The risk assessment was performed and, according to the TTC approach, the obtained results showed that there was no risk associated with the release of the identified substances. However, complementary studies related to the toxicity of the unidentified substances and the potential mixture toxicity (cocktail effects) should be conducted in parallel using bioassays.

## 1. Introduction

In Europe, plastic materials and articles intended to come into contact with food should comply with the Commission Regulation (EU) No 10/2011 [1], which contains the ‘Union list of authorized monomers, other starting substances, macromolecules obtained from microbial fermentation, additives and polymer production aids’ (intentionally added substances, IAS) that can be used for the manufacture of plastic FCM [1]. Moreover, overall and specific migration limits (SML) have also been established [1]. However, during the manufacturing processes and uses of plastic FCM, the reaction and degradation of products can occur (non-intentionally added substances, NIAS). For this reason, the risk associated with the presence and potential release of NIAS should be assessed before the authorization of FCM [2].

In this sense, the non-target analysis of unknown compounds (IAS/NIAS) in plastic FCM is an analytical field that has gained popularity during the last few years [3,4], as demonstrated by the several review articles published recently on this topic [5,6,7,8,9,10].

Regarding the most common analytical techniques, most of the published papers dealing with the non-target analysis of plastic FCM are based on liquid chromatography (LC) coupled to high-resolution mass spectrometry (HRMS) using hybrid mass analyzers, such as quadrupole–time-of-flight (Q-TOF) [11,12] or Q-Orbitrap [4,13,14,15,16]. In this regard, low-energy ionization sources, such as electrospray ionization (ESI), allow observation of the molecular ion, thus easing significantly the identification of substances.

If these analyzers are not used, then published papers that used gas chromatography (GC) coupled to mass spectrometry (MS) usually required tedious and time consuming manual processing of the acquired data in order to perform the identification of the unknown substances [12,17,18,19,20], even being necessary to compare the MS spectra of the acquired peaks one by one with those included in the NIST Mass Spectral Library [21]. In this regard, comprehensive two-dimensional gas chromatography (GC × GC) can also be a useful technique in order to improve the characterization power of GC separations [22].

In general terms, non-target GC analysis enables the characterization of the less polar and more volatile migrant substances, while LC is more suitable for the analysis of polar and non-volatile migrant substances [12,17]. In this respect, the deconvolution of electron ionization (EI) spectra and automated substance identification are major challenges for non-target GC analysis. With regard to MS detectors coupled to GC, most of the published papers have used low-resolution MS [12,20,23,24,25,26], and only a few of them used HRMS detectors, mainly Q-TOF [11,18,27,28] and, less frequently, Orbitrap [19]. In this regard, HRMS offers broad advantages over low-resolution MS for the identification of unknown substances as the molecular formula of the acquired ions can be reliably obtained from their exact mass, thus easing their characterization [29].

After the unknown substances have been detected and tentatively identified, it is necessary to perform a risk assessment to evaluate the safety of the plastic FCM. For substances with no toxicological data available, the threshold of toxicological concern (TTC) approach based on Cramer rules [30,31,32] was applied using Toxtree software [33]. According to Commission Regulation (EU) No 10/2011, NIAS should not migrate into food or food simulants in levels higher than 0.01 mg kg^−1^, except for substances whose genotoxicity has not been discarded, which should not migrate in levels higher than 0.00015 mg kg^−1^ [1]. Moreover, for authorized substances (IAS) according to Commission Regulation (EU) No 10/2011 [1], SML were considered.

According to the European Commission, the demand for recycled plastics is only about the 6% of the total plastic consumption in Europe [34]. The massive production, use, and disposal of plastic packaging are generating enormous amounts of waste and contributing to environmental problems of great concern. For this reason, the research and development of new FCM based on recycled plastic is a matter of interest and an economic field with great potential for plastic and packaging industries.

Among recycled plastic materials, recycled low-density polyethylene (LDPE) is one of the most demanded in a wide variety of applications that require flexible materials, such as carrier bags or shrink and stretch films [35]. However, post-consumer LDPE may contain residues and contaminants from previous uses, non-authorized substances, or substances from non-food applications. For this reason, an exhaustive evaluation of migrant substances, both IAS and NIAS, is necessary in order to ensure the safety of recycled LDPE [1,4,36]. In Europe, recycled plastic FCM need to be manufactured according to recycling processes previously authorized by the European Food Safety Authority (EFSA), in order to ensure that the plastic input originates from plastic materials and articles that have been manufactured in accordance with EU legislation on plastic food contact materials and articles, and that the recycling process eliminates contamination or reduces it to a concentration that does not pose a risk to human health [2,34,36].

The non-target analysis of recycled LDPE, which could be used as FCM, has only been previously considered by our research group, using an LC-HRMS approach [4]. In this sense, the present study constitutes a novel and complementary methodology to perform the identification and risk assessment of the more volatile and less polar migrant substances, which could not be identified with the previously addressed LC approach [4].

To the best of our knowledge, this is the first study addressing a reliable and automated methodology to perform the identification and risk assessment of unknown substances (IAS/NIAS) in recycled LDPE, which could be used as FCM, by GC-Q-Orbitrap HRMS.

## 2. Materials and Methods

### 2.1. Reagents and Samples

Analytical standards of Benzophenone (CAS 119-61-9), Butyl stearate (CAS 123-95-5), Diisobutyl phthalate (CAS 84-69-5), and Tris (2,4-di-tert-butylphenyl) phosphite (Irgafos 168, CAS 31570-04-4), from LGC Standards (Bury, United Kingdom), and Bis (2-ethylhexyl) adipate (CAS 103-23-1), Bis (2-ethylhexyl) phthalate (CAS 117-81-7), Bis (2-ethylhexyl) terephthalate (CAS 6422-86-2), Butylated hydroxytoluene (CAS 128-37-0), Diphenyl sulphone (CAS 127-63-9), and Tri-n-butyl acetyl citrate (CAS 77-90-7), from Merck KGaA (Darmstadt, Germany), were used as standards for confirmation and quantification.

Analytical standards of Phenol-13C6 (CAS 89059-34-7), Benzophenone-D10 (CAS 22583-75-1), and Bis (2-ethylhexyl) phthalate-D4 (CAS 93951-87-2), all from LGC Standards (Bury, UK), were used as internal standards.

Standard n-alkane mixtures, C8-C20 and C10-C40 (all even), both from Merck KGaA (Darmstadt, Germany), were used for retention index calculation.

Acetone ≥ 99.8%, residue-analysis grade, from VWR International (Radnor, PA, USA), was used as solvent.

The analyzed sample was a post-consumer recycled LDPE film provided by Cadel Deinking, S.L. (Sant Vicent del Raspeig, Spain). The recycled LDPE film was obtained through a patented process that uses water-based chemicals to remove contaminants and includes grinding, washing, drying, and extrusion [37].

### 2.2. Analytical Strategy

**Figure 1:**
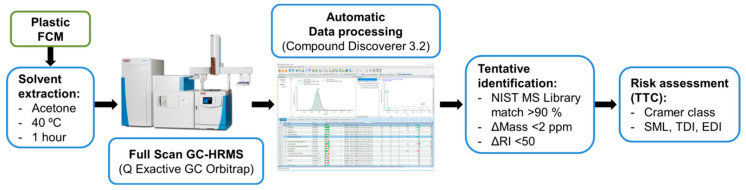
Scheme of the overall analytical methodology.

The developed analytical strategy, which includes solvent extraction of the plastic FCM, GC-HRMS analysis, automated data processing, and tentative compound identification, is depicted in Figure 1.

#### 2.2.1. Sample Preparation and Solvent Extraction

The recycled LDPE sample was extracted with an organic solvent in order to release the contaminants from the polymeric matrix. In this case, acetone was selected as extraction solvent to simulate the migration of unknown substances (IAS/NIAS). According to Commission Regulation (EU) No 10/2011 [1], migration tests should be performed using food simulants under standardized conditions. With regard to the food simulants described in Commission Regulation (EU) No 10/2011 [1], in the case of aqueous and hydrophilic foods, ethanol–water mixtures, i.e., food simulants A, C, and D1 (with an ethanol content of 10, 20, and 50% *v/v*, respectively), and food simulant B (acetic acid 3%, *w/v*), in the case of acid foods, are proposed to perform migration tests. In the case of fatty foods, the regulated food simulants are vegetable oil (food simulant D2) or alternative food simulants, such as ethanol 95% or iso-octane. However, a direct extraction of the plastic sample with an organic solvent was preferred as a faster and more extractive procedure. Among the different organic solvents that could be used for this purpose, acetone was selected due to its high volatility and low boiling point (56 °C), it being a suitable solvent for direct injection into the GC system, its moderate polarity (2.69 D), it being able to extract a wide range of polar and non-polar substances, and its low toxicity, compared to other possible organic solvents, such as n-hexane or dichloromethane.

Compared to the regulated food simulants, acetone presents a higher extraction capability than ethanol–water mixtures in order to extract both polar and non-polar compounds, and it is similar to the alternative food simulants for fatty foods with regard to the extraction of less polar substances. For that reason, acetone was selected as a compromise situation to cover any possible application of the recycled FCM, for both aqueous and/or fatty foods. With regard to test conditions, 1 h of extraction time was selected as a faster approach compared to the standardized conditions of Commission Regulation (EU) No 10/2011 [1], which usually range from 24 h to 10 days.

By triplicate, a portion of 5 cm × 5 cm (0.25 dm^2^) of the recycled LDPE film was introduced in a 50 mL glass beaker with 20 mL of acetone and kept in an oven at 40 °C for 1 h. The beaker was sealed with aluminum foil to prevent solvent evaporation. After that, the whole sample extract was collected using only glassware material and it was evaporated under gently nitrogen stream to 0.5 mL. Then, the concentrated extract was transferred to a 1 mL volumetric flask, spiked with 200 ng mL^−1^ of the internal standards, and filled up to the line using the same solvent. Finally, the sample extract was placed into an injection vial for GC analysis. Additionally, an extraction blank was prepared by triplicate following the same procedure, leaving out the recycled LDPE sample. In order to prevent contamination from the laboratory environment, all the glassware materials were rinsed with acetone and drained before their use.

#### 2.2.2. GC-HRMS Analysis

A Trace 1310 GC system equipped with a TraceGOLD TG-5MS column (30 m, 0.25 mm, 0.25 µm), coupled to a Q-Exactive GC Orbitrap HRMS detector, all from Thermo Fisher Scientific (Waltham, MA, USA), were used. The injection volume was 1 µL (splitless mode) and the inlet was set at 280 °C. The GC operated in constant flow mode at 1.2 mL min^−1^ of helium as carrier gas, using the following oven temperature program: 40 °C, held for 5 min; 5 °C min^−1^ up to 315 °C, held for 10 min. The MS transfer line was set at 300 °C. The EI ion source operated at 70 eV, and the ion source temperature was set at 250 °C. The acquisition was performed in full scan mode with a resolving power of 60,000 FWHM and a mass range from 40 to 500 *m/z*.

To perform retention index (RI) calculation, standard n-alkane mixtures, C8-C20 and C10-C40 (all even), were injected with the same conditions.

#### 2.2.3. Data Processing for Tentative Identification

In order to perform the tentative identification of the acquired features, the obtained data were processed automatically using the software Compound Discoverer™ 3.2 (CD 3.2) [38], from Thermo Fisher Scientific (Waltham, MA, USA). Briefly, the automatic data processing workflow performed alignment of retention time, deconvolution of EI spectra, unknown compound identification, and removal of background features. CD 3.2 automatically identifies substances using NIST Mass Spectral Library [21] and local database Mass Lists searches (in the present study, the ‘Extractable and Leachable HRMS database’ containing 1741 compounds, the ‘GC Orbitrap Contaminants library’ containing 880 compounds, the ‘GC Orbitrap Flavor and Fragrance database’ containing 49 compounds, the ‘GC Orbitrap Metabolomics library’ containing 1014 compounds, and a home-made database containing 674 plastic-related substances).

For the tentative identification of the acquired features, a restrictive set of identification criteria, including EI spectra match with NIST Mass Spectral Library, exact mass of annotated fragments, and retention index (RI), was used. These parameters are shown in Table 1.

As can be seen, in order to consider a proposed substance by CD 3.2 as tentatively identified, a positive match (Total score) > 90% with NIST Mass Spectral Library was required. ‘Total score’ is a composite metric that includes contribution from the ‘High resolution filtering score’ (HRF) and the ‘Search index’ (SI) score. According to this, a higher ‘Total score’ implies a higher probability of a positive match for a proposed substance. In addition to that, an exact mass accuracy (ΔMass) < 2 ppm for at least 3 annotated fragments (matching fragment ions between acquired spectrum and NIST library spectrum), or 2 fragments and the molecular ion, if observed, was also required. With regard to retention index (RI), an absolute difference (ΔRI) < 50 units between calculated RI and NIST library RI (column type: semi standard non polar) was required. In cases where the RI value was not available in the NIST library for the proposed substances, the most probable compound according to the abovementioned criteria was considered. All these parameters were calculated and provided automatically by CD 3.2 software.

Moreover, CD 3.2 also performed searches on local database Mass Lists, which include structure, molecular formula, and exact mass of substances. Despite EI spectra not being included in Mass Lists, they are useful to increase the confidence of the tentative identification if a positive match with a Mass List is obtained and the molecular ion of the proposed substance is also observed.

### 2.3. Risk Assessment

To perform the risk assessment of the released substances, the threshold of toxicological concern (TTC) approach was applied by using the Toxtree software [33]. This approach estimates the tolerable daily intake (TDI, mg person^−1^ day^−1^) for a given substance through the Cramer decision rules, according to molecular structure, which assign each substance into one Cramer class of toxicological hazard: Class I (Low hazard), 1.80 mg person^−1^ day^−1^; Class II (Intermediate hazard), 0.54 mg person^−1^ day^−1^; Class III (High hazard), 0.09 mg person^−1^ day^−1^. After that, TDI values were compared with the estimated daily intake (EDI, mg person^−1^ day^−1^), calculated considering the current European default assumption that a reference adult person consumes one kilogram of packed food per day [39], according to the following expression: EDI (mg person^−1^ day^−1^) = Migration (mg kg^−1^) × 1 kg (daily intake of packed food). Migration (mg kg^−1^) was estimated considering the average response factor of internal standards [2]. The average response factor of internal standards was obtained as the average ratio between the peak area of the internal standard and its known added concentration. From that, the estimated concentrations of unknown substances were obtained as the ratio between their peak area and the average response factor. In order to express the values of estimated migration in mg kg^−1^, the conventional food contact surface ratio of 6 dm^2^ per kg of food was considered [1].

According to Commission Regulation (EU) No 10/2011 [1], a maximum limit of 0.01 mg kg^−1^ is established for the migration of NIAS from FCM across a functional barrier. In the case of substances for which genotoxicity has not been discarded, a maximum limit of 0.00015 mg kg^−1^ is applicable [1]. For this reason, only the identified substances with an estimated migration over 0.00015 mg kg^−1^ were considered for the risk assessment.

## 3. Results and Discussion

### 3.1. System Suitability

In order to evaluate the system suitability of the GC-HRMS analysis, a standard solution containing 200 ng mL^−1^ of the internal standards was analyzed in triplicate before and after the acquisition workflow. The system suitability was evaluated in terms of relative standard deviation (RSD, %) of base peak area and retention time, and mass accuracy (ΔMass, ppm) of the internal standards. The RSD of base peak area ranged from 2.4 to 8.3%, and RSD of retention time ranged from 0.02 to 0.1%. Mass accuracy ranged from −0.13 to 0.74 ppm, thus showing that the allowed mass accuracy of 2 ppm for the identification criteria (see Table 1) is wider enough to avoid false negatives. These results show that the GC-HRMS system operated steadily and accurately during the analytical sequence.

### 3.2. Identification of Unknown Substances

After the recycled LDPE sample was analyzed in triplicate, the acquired data were processed using CD 3.2 as specified above (see Section 2.2.3). The software automatically annotated 374 features, of which 83 compounds could be tentatively identified after examination of the identification criteria (see Table 1). The 83 identified substances are shown in Table 2. It is necessary to consider that the post-consumer recycled LDPE could present contaminants from a wide variety of sources, such as residues from previous uses, or reaction and degradation products during the manufacture and recycling processes.

As stated above, all compounds presented a positive match (Total score) > 90% with NIST Mass Spectral Library [21], an exact mass accuracy (ΔMass) < 2 ppm for at least 3 annotated fragments, or 2 fragments and the molecular ion, if observed, and an absolute RI difference (ΔRI) < 50 units between calculated RI and NIST library RI, if available.

Out of 83 tentatively identified substances, only 12 are included in the ‘Union list of authorized substances’ (IAS) of Commission Regulation 10/2011 [1]. As can be seen, most of the identified substances were polymer additives, such as plasticizers, surfactants, stabilizers, or emulsifiers. Moreover, some metabolites and substances with a wide variety of industrial, chemical, and cosmetic applications were also identified. In addition to that, different linear and branched polyethylene oligomers (C_n_H_2n+2_) were found in the analyzed LDPE sample. Many of the identified short-chain linear oligomers present industrial, chemical, and cosmetic applications, so they can be present as contaminants. However, their presence can also be due to the partial degradation of LDPE during the recycling process or the solvent extraction. In this respect, the degradation of the recycled LDPE during the solvent extraction process, although undesired, can facilitate the release of contaminants from the polymeric matrix.

The other 291 unidentified features did not meet the necessary criteria to be tentatively identified with a sufficient level of confidence (see Table 1). On the other hand, the used mass range (from 40 to 500 *m/z*) may have meant that substances with fragment ions above 500 *m/z* were not detected or underestimated. A wider range could be used if allowed by the instrument characteristics.

### 3.3. Confirmation with Standards

In order to validate the proposed methodology, commercially available analytical standards of 10 of the 83 tentatively identified substances were purchased (see Section 2.1), and they were used to confirm their identity and to quantify them. Standard solutions containing the analytes from 20 to 500 ng mL^−1^ and 200 ng mL^−1^ of the internal standards were prepared and analyzed with the same conditions (see Section 2.2.2).

**Figure 2:**
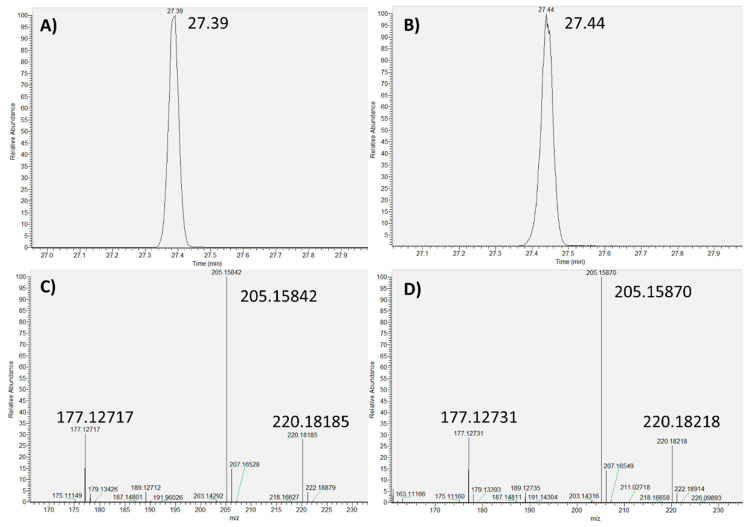
Butylated hydroxytoluene (BHT): (**A**) Base peak (standard); (**B**) Base peak (sample); (**C**) MS spectrum (standard); (**D**) MS spectrum (sample).

**Figure 3:**
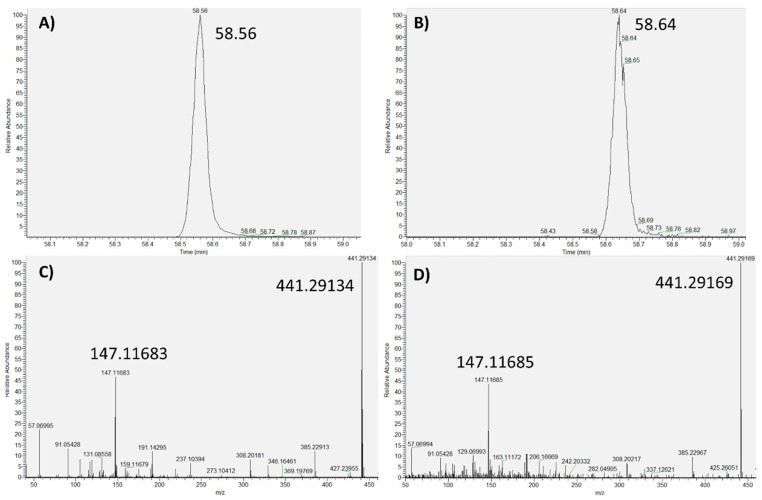
Tris (2,4-di-tert-butylphenyl) phosphite (Irgafos 168): (**A**) Base peak (standard); (**B**) Base peak (sample); (**C**) MS spectrum (standard); (**D**) MS spectrum (sample).

The tested compounds were evaluated in terms of the acquired retention time, exact mass, and MS spectrum, between the analyzed LDPE sample and the standards. As an example, the chromatographic base peak and the MS spectrum, acquired in the standard solution and in the analyzed sample, for Butylated hydroxytoluene (BHT) and Tris (2,4-di-tert-butylphenyl) phosphite (Irgafos 168) are shown in Figure 2 and Figure 3, respectively.

All of the tested compounds presented a good match between the acquired features in the analyzed LDPE sample and the standards, thus showing that the proposed methodology provided reliable results. It can be noted that a slight drift in retention time was observed due to the elapsed time between the sample analysis and the confirmation with standards. However, this drift did not significantly affect the obtained results.

It should be noted that, although quantification was performed for 10 of the tentatively identified substances, the objective of the present study is not to provide a target quantitative procedure for these or other particular substances, but to provide a reliable methodology that can be used as a screening tool to perform the identification and risk assessment of unknown substances in plastic FCM. In this sense, these 10 analytical standards were selected due to their commercial availability in order to validate the proposed strategy for the tentative identification of unknown substances.

### 3.4. Risk Assessment of Recycled LDPE

After the tentative identification of the detected substances by applying the proposed methodology (see Section 2.2), a risk assessment was performed using the TTC approach (see Section 2.3). According to Commission Regulation (EU) No 10/2011 [1], a maximum limit of 0.01 mg kg^−1^ is established for the migration of NIAS from FCM across a functional barrier, except for substances whose genotoxicity has not been discarded, which should not migrate in levels higher than 0.00015 mg kg^−1^. In this sense, only the identified substances with an estimated migration over 0.00015 mg kg^−1^ were considered for the risk assessment. Out of 83 identified substances, only 9 were found at a migration level above 0.01 mg kg^−1^, and 45 substances were found at a migration level above 0.00015 mg kg^−1^. Some of the assessed compounds were IAS, according to the abovementioned regulation. In those cases, their specific migration limits (SML) were considered. These results are shown in Table 3.

As can be seen, all of the identified NIAS presented an estimated daily intake (EDI) lower than their tolerable daily intake (TDI), according to their toxicological hazard (Cramer class). Moreover, all of the identified IAS presented a migration level lower than their SML. Therefore, it can be concluded that there was no risk associated with the release of the identified substances from the recycled LDPE film. Moreover, it would be necessary to perform further studies in order to tentatively identify and to evaluate the risk associated with the release of the 291 unidentified substances detected in the analyzed LDPE sample, which could present potential genotoxicity. Likewise, toxicity studies related with the unidentified substances and mixture toxicity (cocktail effects) would be necessary.

Although substance migration was not performed using food simulants, according to Commission Regulation (EU) No 10/2011 [1], the methodology presented in this paper constitutes a faster and reliable procedure to perform a first screening approach to assess the safety of plastic FCM after direct solvent extraction.

## 4. Conclusions

In this work, a fast and automatic strategy has been developed for the non-target analysis of plastic materials and articles intended to come into contact with food (FCM) by GC-Q-Orbitrap HRMS, including tentative identification of unknown substances (IAS/NIAS) and risk assessment by using the threshold of toxicological concern (TTC) approach.

As a proof-of-concept demonstration, the proposed methodology was applied to post-consumer recycled LDPE film, which could be used as FCM, obtaining 374 annotated features, of which 83 were tentatively identified after examination of the identification criteria. Moreover, 10 of these were successfully confirmed with commercial analytical standards, thus showing that the proposed methodology provided reliable results. Most of the identified compounds were plastic additives, such as plasticizers, used in different plastic applications. Moreover, oligomers of LDPE, metabolites, substances with industrial, chemical, and cosmetic applications, and other plastic-related substances were also identified. After performing the tentative identification, a risk assessment of the analyzed LDPE was carried out, showing that the release of the identified substances did not represent a safety risk. However, complementary studies related with the toxicity of the unidentified substances and the potential mixture toxicity (cocktail effects) should be conducted in parallel using bioassays.

The proposed analytical methodology, including solvent extraction, GC-HRMS analysis, tentative identification, and risk assessment constitutes a fast and automatic approach that can be applied for the non-target analysis of unknown substances (IAS/NIAS) of different plastic FCM, showing its great utility and versatility.

## Figures and Tables

**Table 1:** Criteria used for the tentative identification of unknown substances.

Parameter	Criteria
NIST MS Library match (Total score) ^1^	>90%
Exact mass accuracy (ΔMass) ^2^	<2 ppm
Retention index absolute difference (ΔRI) ^3^	<50 units

^1^ Match between deconvoluted EI spectrum and NIST Mass Spectral Library [21]. Total score is a composite metric that includes contribution from the High resolution filtering score (HRF) and the Search index (SI) score. ^2^ Exact mass accuracy (ΔMass) for at least 3 annotated fragments or 2 fragments and molecular ion, when observed. ^3^ Retention index absolute difference (ΔRI) between calculated RI and NIST library RI.

**Table 2:** Tentatively identified substances in the analyzed recycled LDPE sample.

Compound Name ^1^	CAS Number	Molecular Formula	NIST Match (Total Score, %) ^2^	ΔRI (a.u.) ^3^
Acetate esters				
7-Tetradecen-1-yl acetate	16974-10-0	C16 H30 O2	93.2	24
Hexadecyl acetate	629-70-9	C18 H36 O2	93.5	3
Hexadecyl trifluoroacetate	6222-03-3	C18 H33 F3 O2	95.9	20
Octadecyl trifluoroacetate	79392-43-1	C20 H37 F3 O2	95.6	14
Tri-n-butyl acetyl citrate *	77-90-7	C20 H34 O8	95.7	4
Aldehydes and ketones				
Benzophenone *	119-61-9	C13 H10 O	95.8	8
2,6-Di-tert-butyl-1,4- benzoquinone	719-22-2	C14 H20 O2	94.9	10
3,5-Di-tert-butyl-4- hydroxybenzaldehyde	1620-98-0	C15 H22 O2	96.1	17
7,9-Di-tert-butyl-1-oxaspiro(4,5)deca-6,9-diene-2,8-dione	82304-66-3	C17 H24 O3	96.1	21
Dehydroabietic aldehyde	13601-88-2	C20 H28 O	96.4	1
Alkenes				
1-Hexadecene	629-73-2	C16 H32	96.5	1
9-Nonadecene	31035-07-1	C19 H38	91.4	29
1-Docosene	1599-67-3	C22 H44	91.3	50
9-Tricosene	27519-02-4	C23 H46	93.5	3
1-Tetracosene	10192-32-2	C24 H48	95.3	0
1-Hexacosene	18835-33-1	C26 H52	93.7	2
Squalene	111-02-4	C30 H50	96.4	0
Phenol derivatives				
1,2-Diphenoxyethane	104-66-5	C14 H14 O2	97.5	10
2,4-Di-tert-butylphenol	96-76-4	C14 H22 O	97.2	12
2,6-Di-tert-butylphenol	128-39-2	C14 H22 O	96.8	10
Butylated hydroxytoluene *	128-37-0	C15 H24 O	97.0	9
2,4-Di-tert-pentylphenol	120-95-6	C16 H26 O	92.6	38
Metilox	6386-38-5	C18 H28 O3	94.7	16
Irganox 1076 *	2082-79-3	C35 H62 O3	93.0	8
Phthalates				
Dibutyl phthalate *	84-74-2	C16 H22 O4	94.6	2
Diisobutyl phthalate	84-69-5	C16 H22 O4	96.4	13
Bis(2-ethylhexyl) phthalate *	117-81-7	C24 H38 O4	98.2	2
Bis(2-ethylhexyl) terephthalate *	6422-86-2	C24 H38 O4	94.7	28
Primary alcohols				
3-Nonenol	10340-23-5	C9 H18 O	95.7	48
Octadecanol	112-92-5	C18 H38 O	96.2	3
Nonadecanol	1454-84-8	C19 H40 O	96.3	18
Eicosanol	629-96-9	C20 H42 O	90.9	20
Docosanol	661-19-8	C22 H46 O	90.4	12
Tetracosanol	506-51-4	C24 H50 O	86.2	11
Cyclic and aromatic hydrocarbons				
2,6-Diisopropylnaphthalene	24157-81-1	C16 H20	96.7	8
2,2′,5,5′-Tetramethyl-1,1′- biphenyl	3075-84-1	C16 H18	94.2	42
Undecylcyclohexane	54105-66-7	C17 H34	94.6	3
1-Ethyldecylbenzene	2400-00-2	C18 H30	90.3	1
7-Isopropyl-1-methyl-1,2,3,4- tetrahydrophenanthrene	6566-19-4	C18 H22	96.2	11
7-Isopropyl-1,4-dimethyl tetradecahydrophenanthrene	2221-95-6	C19 H34	96.7	9
m-Camphorene	20016-73-3	C20 H32	92.6	44
1,3,5-Triphenylcyclohexane	28336-57-4	C24 H24	95.3	31
Fatty acid methyl esters (FAMEs)				
Methyl laurate	111-82-0	C13 H26 O2	95.8	13
Methyl palmitate	112-39-0	C17 H34 O2	97.7	16
Methyl heptadecanoate	1731-92-6	C18 H36 O2	94.2	11
Methyl linolelaidate	2566-97-4	C19 H34 O2	98.0	20
Methyl elaidate	1937-62-8	C19 H36 O2	98.1	4
Methyl stearate	112-61-8	C19 H38 O2	97.8	12
Methyl erucate	1120-34-9	C23 H44 O2	91.8	0
Methyl isopimarate	1686-62-0	C21 H32 O2	92.1	1
Methyl icosanoate	1120-28-1	C21 H42 O2	97.2	13
Other fatty acid esters (FAEs)				
Isopropyl myristate	110-27-0	C17 H34 O2	94.3	4
Ethyl palmitate	628-97-7	C18 H36 O2	92.1	1
Butyl palmitate *	111-06-8	C20 H40 O2	93.1	4
Butyl stearate *	123-95-5	C22 H44 O2	94.3	3
Hexadecyl palmitate	540-10-3	C32 H64 O2	95.0	0
Linear and branched polyethylene oligomers				
Tetradecane	629-59-4	C14 H30	96.5	0
Hexadecane	544-76-3	C16 H34	96.7	0
Heptadecane	629-78-7	C17 H36	96.9	2
Octadecane	593-45-3	C18 H38	96.6	0
Eicosane	112-95-8	C20 H42	96.5	0
Tetracosane	646-31-1	C24 H50	95.7	0
Pentacosane	629-99-2	C25 H52	94.0	31
Heptacosane	593-49-7	C27 H56	94.4	2
Octacosane	630-02-4	C28 H58	96.1	0
Tetraiacontane	14167-59-0	C34 H70	92.1	0
3-Methylpentadecane	2882-96-4	C16 H34	93.1	1
3-Methylheptadecane	6418-44-6	C18 H38	94.9	2
3-Methylnonadecane	6418-45-7	C20 H42	95.5	2
2,6,10,15-Tetramethyl heptadecane	54833-48-6	C21 H44	92.3	30
3-Methylheneicosane	6418-47-9	C22 H46	95.9	1
5-Methylheneicosane	25117-37-7	C22 H46	95.5	1
11-Methyltricosane	27538-41-6	C24 H50	95.3	38
2-Methyloctacosane	1560-98-1	C29 H60	95.7	43
5-Methylnonacosane	71868-29-6	C30 H62	94.1	9
Other compounds				
Diphenyl sulphone *	127-63-9	C12 H10 O2 S	93.0	-
1-Chlorohexadecane	4860-03-1	C16 H33 Cl	94.2	21
N,N-Dimethylpalmitylamine	112-69-6	C18 H39 N	96.1	-
Galaxolide	1222-05-5	C18 H26 O	95.8	-
Methyl dehydroabietate	1235-74-1	C21 H30 O2	97.6	2
Methyl abietate	127-25-3	C21 H32 O2	96.2	4
Bis(2-ethylhexyl) adipate *	103-23-1	C22 H42 O4	93.5	12
Irgafos 168 *	31570-04-4	C42 H63 O3 P	94.7	5

^1^ All compounds presented an exact mass accuracy (ΔMass) <2 ppm for at least 3 fragment ions, or 2 fragments and molecular ion. ^2^ Exact mass accuracy (ΔMass) for at least 3 annotated fragments or 2 fragments and molecular ion, when observed. ^3^ Retention index absolute difference (ΔRI) between calculated RI and NIST library RI. * Authorized substances (IAS) according to Commission Regulation (EU) No 10/2011 [1].

**Table 3:** Risk assessment of the identified substances.

Compound Name	CAS Number	Migration (mg kg^−1^) ^1^	SML (mg kg^−1^) ^2^	Toxicological Hazard (Cramer Class) ^3^	TDI (mg Person^−1^ day^−1^) ^4^	EDI (mg Person^−1^ day^−1^) ^5^
Irgafos 168 *	31570-04-4	2.0 ± 0.2	-			
Methyl palmitate	112-39-0	0.058		Low (Class I)	1.80	0.058
Methyl stearate	112-61-8	0.058		Low (Class I)	1.80	0.058
1,2-Diphenoxyethane	104-66-5	0.033		High (Class III)	0.09	0.033
Octadecane	593-45-3	0.021		Low (Class I)	1.80	0.021
Bis(2-ethylhexyl) terephthalate *	6422-86-2	0.020 ± 0.002	60			
Tetracosane	646-31-1	0.018		Low (Class I)	1.80	0.018
Octacosane	630-02-4	0.013		Low (Class I)	1.80	0.013
Butylated hydroxytoluene *	128-37-0	0.010 ± 0.001	3			
Hexadecane	544-76-3	0.009		Low (Class I)	1.80	0.009
Methyl dehydroabietate	1235-74-1	0.009		Low (Class I)	1.80	0.009
Tetraiacontane	14167-59-0	0.007		Low (Class I)	1.80	0.007
Eicosane	112-95-8	0.005		Low (Class I)	1.80	0.005
Benzophenone *	119-61-9	0.0030 ± 0.0005	0.6			
Bis(2-ethylhexyl) phthalate *	117-81-7	0.0025 ± 0.0005	1.5			
2,6-Di-tert-butylphenol	128-39-2	0.002		Intermediate (Class II)	0.54	0.002
N,N-Dimethylpalmitylamine	112-69-6	0.002		Low (Class I)	1.80	0.002
Octadecyl trifluoroacetate	79392-43-1	0.0014		High (Class III)	0.09	0.0014
2,6-Diisopropylnaphthalene	24157-81-1	0.0014		High (Class III)	0.09	0.0014
Diisobutyl phthalate	84-69-5	0.0014 ± 0.0002		Low (Class I)	1.80	0.0014
Irganox 1076 *	2082-79-3	0.0013	6			
7,9-Di-tert-butyl-1-oxaspiro(4,5)deca-6,9-diene-2,8-dione	82304-66-3	0.0010		High (Class III)	0.09	0.0010
Methyl linolelaidate	2566-97-4	0.0009		Low (Class I)	1.80	0.0009
1-Tetracosene	10192-32-2	0.0009		Low (Class I)	1.80	0.0009
2,4-Di-tert-pentylphenol	120-95-6	0.0008		Low (Class I)	1.80	0.0008
Methyl elaidate	1937-62-8	0.0007		Low (Class I)	1.80	0.0007
Nonadecanol	1454-84-8	0.0007		Low (Class I)	1.80	0.0007
Squalene	111-02-4	0.0007		Low (Class I)	1.80	0.0007
3,5-Di-tert-butyl-4- hydroxybenzaldehyde	1620-98-0	0.0005		Intermediate (Class II)	0.54	0.0005
2,4-Di-tert-butylphenol	96-76-4	0.0005		Low (Class I)	1.80	0.0005
2,6-Di-tert-butyl-1,4- benzoquinone	719-22-2	0.0005		Intermediate (Class II)	0.54	0.0005
Bis(2-ethylhexyl) adipate *	103-23-1	0.0005 ± 0.0001	18			
Galaxolide	1222-05-5	0.0005		High (Class III)	0.09	0.0005
1-Chlorohexadecane	4860-03-1	0.0004		High (Class III)	0.09	0.0004
1-Hexadecene	629-73-2	0.0003		Low (Class I)	1.80	0.0003
Hexadecyl palmitate	540-10-3	0.0003		Low (Class I)	1.80	0.0003
Isopropyl myristate	110-27-0	0.0003		Low (Class I)	1.80	0.0003
Hexadecyl trifluoroacetate	6222-03-3	0.0003		High (Class III)	0.09	0.0003
Methyl abietate	127-25-3	0.0003		High (Class III)	0.09	0.0003
2,6,10,15-Tetramethyl heptadecane	54833-48-6	0.0003		Low (Class I)	1.80	0.0003
Butyl palmitate *	111-06-8	0.0002	-			
11-Methyltricosane	27538-41-6	0.0002		Low (Class I)	1.80	0.0002
Hexadecyl acetate	629-70-9	0.0002		Low (Class I)	1.80	0.0002
3-Nonenol	10340-23-5	0.00018		Low (Class I)	1.80	0.00018
Metilox	6386-38-5	0.00017		Intermediate (Class II)	0.54	0.00017

^1^ Migration (mg kg^−1^) estimated from the average response factor of internal standards or determined with analytical standards. ^2^ Specific migration limit (SML) according to Commission Regulation (EU) No 10/2011 [1]. ^3^ Cramer class of toxicological hazard calculated using Toxtree software [33]. ^4^ Tolerable daily intake (TDI) value depending on toxicological hazard (Cramer class). ^5^ Estimated daily intake (EDI) value calculated from Migration (mg kg^−1^) according to current European default assumption [39]. * Authorized substances (IAS) according to Commission Regulation (EU) No 10/2011 [1].

## Data Availability

Data are available upon reasonable request from the corresponding author.

## References

[1] Regulation C. No 10/2011 of 14 January 2011 on Plastic Materials and Articles Intended to Come into Contact with Food (and Its Successive Amendments). http://data.europa.eu/eli/reg/2011/10/2020-09-23.

[2] International Life Science Institute (ILSI) Europe (2015). Guidance on Best Practices on the Risk Assessment of Non-Intentionally Added Substances (NIAS) in Food Contact Materials and Articles.

[3] Aznar M., Alfaro P., Nerín C., Jones E., Riches E. (2016). Progress in mass spectrometry for the analysis of set-off phenomena in plastic food packaging materials. J. Chromatogr. A.

[4] Yusà V., López A., Dualde P., Pardo O., Fochi I., Pineda A., Coscolla C. (2020). Analysis of unknowns in recycled LDPE plastic by LC-Orbitrap Tribrid HRMS using MS^3^ with an intelligent data acquisition mode. Microchem. J..

[5] Gallart-Ayala H., Nunez O., Lucci P. (2013). Recent advances in LC-MS analysis of food-packaging contaminants. TrAC Trends Anal. Chem..

[6] Hoppe M., de Voogt P., Franz R. (2016). Identification and quantification of oligomers as potential migrants in plastics food contact materials with a focus in polycondensates—A review. Trends Food Sci. Technol..

[7] Sanchis Y., Yusà V., Coscollà C. (2017). Analytical strategies for organic food packaging contaminants. J. Chromatogr. A.

[8] Martínez-Bueno M.J., Gómez Ramos M.J., Bauer A., Fernández-Alba A.R. (2019). An overview of non-targeted screening strategies based on high resolution accurate mass spectrometry for the identification of migrants coming from plastic food packaging materials. TrAC Trends Anal. Chem..

[9] Wrona M., Nerín C. (2020). Analytical approaches for analysis of safety of modern food packaging: A review. Molecules.

[10] Ouyang X., Lu Z., Hu Y., Xie Z., Li G. (2021). Research progress on sample pretreatment methods for migrating substances from food contact materials. J. Sep. Sci..

[11] Martínez-Bueno M.J., Cimmino S., Silvestre C., Tadeo J.L., García-Valcárcel A.I., Fernández-Alba A.R., Hernando M.D. (2016). Characterization of non-intentionally added substances (NIAS) and zinc oxide nanoparticle release from evaluation of new antimicrobial food contact materials by both LC-QTOF-MS, GC-QTOF-MS and ICP-MS. Anal. Methods.

[12] Canellas E., Vera P., Nerín C. (2015). UPLC-ESI-Q-TOF-MS^E^ and GC-MS identification and quantification of non-intentionally added substances coming from biodegradable food packaging. Anal. Bioanal. Chem..

[13] Liu A., Qu G., Zhang C., Gao Y., Shi J., Du Y., Jiang G. (2015). Identification of two novel brominated contaminants in water samples by ultra-high performance liquid chromatography-Orbitrap Fusion Tribrid mass spectrometer. J. Chromatogr. A.

[14] Wu Y., Gao S., Liu Z., Zhao J., Ji B., Zheng X., Yu Z. (2019). The quantification of chlorinated paraffins in environmental samples by ultra-high-performance liquid chromatography coupled with Orbitrap Fusion Tribrid mass spectrometry. J. Chromatogr. A.

[15] Yusà V., López A., Dualde P., Pardo O., Fochi I., Miralles P., Coscollà C. (2021). Identification of 24 unknown substances (IAS/NIAS) from food contact polycarbonate by LC-Orbitrap Tribrid HRMS-DDMS^3^: Safety assessment. Int. J. Anal. Chem..

[16] Miralles P., López A., Dualde P., Coscollà C., Yusà V. (2021). LC-Orbitrap Tribrid high-resolution MS using data dependent-tandem mass spectrometry with triple stage fragmentation as a screening tool to perform identification and risk assessment of unknown substances in food contact epoxy resin. J. Sep. Sci..

[17] Horodytska O., Cabanes A., Fullana A. (2020). Non-intentionally added substances (NIAS) in recycled plastics. Chemosphere.

[18] Canellas E., Vera P., Nerín C. (2014). Atmospheric pressure gas chromatography coupled to quadrupole-time of flight mass spectrometry as a tool for identification of volatile migrants from autoadhesive labels used for direct food contact. J. Mass Spectrom..

[19] Martínez-Bueno M.J., Hernando M.D., Uclés S., Rajski L., Cimmino S., Fernández-Alba A.R. (2017). Identification of non-intentionally added substances in food packaging nano films by gas and liquid chromatography coupled to orbitrap mass spectrometry. Talanta.

[20] García Ibarra V., Rodríguez Bernaldo de Quirós A., Paseiro Losada P., Sendón R. (2019). Non-target analysis of intentionally and non-intentionally added substances from plastic packaging materials and their migration into food simulants. Food Packag. Shelf Life.

[21] National Institute of Standards and Technology (NIST) (2020). NIST/EPA/NIH Mass Spectral Library (NIST 20).

[22] Biedermann M., Grob K. (2015). Comprehensive two-dimensional gas chromatography for characterizing mineral oils in foods and distinguishing them from synthetic hydrocarbons. J. Chromatogr. A.

[23] Franz R., Welle F. (2020). Contamination levels in recollected PET bottles from non-food applications and their impact on the safety of recycled PET for food contact. Molecules.

[24] Van Velzen E.U.T., Brouwer M.T., Stärker C., Welle F. (2020). Effect of recycled content and rPET quality of the properties of PET bottles, part II: Migration. Packag. Technol. Sci..

[25] Omer E., Bichon E., Hutinet S., Royer A., Monteau F., Germon H., Hill P., Remaud G., Dervilly-Pinel G., Cariou R. (2019). Toward the characterisation of non-intentionally added substances migrating from polyester-polyurethane lacquers by comprehensive gas chromatography-mass spectrometry technologies. J. Chromatogr. A.

[26] Song X., Wrona M., Nerin C., Lin Q., Zhong H. (2019). Volatile non-intentionally added substances (NIAS) identified in recycled expanded polystyrene containers and their migration into food simulants. Food Packag. Shelf Life.

[27] Canellas E., Vera P., Domeño C., Alfaro P., Nerín C. (2012). Atmospheric pressure gas chromatography coupled to quadrupole-time of flight mass spectrometry as a powerful tool for identification of non-intentionally added substances in acrylic adhesives used in food packaging materials. J. Chromatogr. A.

[28] Ubeda S., Aznar M., Nerín C. (2019). Determination of volatile compounds and their sensory impact in a biopolymer based on polylactic acid (PLA) and polyester. Food Chem..

[29] Onghena M., van Hoeck E., van Loco J., Ibáñez M., Cherta L., Portolés T., Pitarch E., Hernández F., Lemière F., Covaci A. (2015). Identification of substances migrating from plastic baby bottles using a combination of low-resolution and high-resolution mass spectrometric analysers coupled to gas and liquid chromatography. J. Mass Spectrom..

[30] EFSA Scientific Committee (2012). Scientific opinion on exploring options for providing advice about possible human health risk based on the concept of threshold of toxicological concern (TTC). EFSA J..

[31] Canellas E., Vera P., Nerin C. (2017). Migration assessment and the ‘threshold of toxicological concern’ applied to the safe design of an acrylic adhesive for food-contact laminates. Food Addit. Contam. Part A Chem. Anal. Control Expo. Risk Assess..

[32] Pieke E.N., Granby K., Teste B., Smedsgaard J., Rivière G. (2018). Prioritization before risk assessment: The viability of uncertain data on food contact materials. Regul. Toxicol. Pharmacol..

[33] Patlewicz G., Jeliazkova N., Safford R.J., Worth A.P., Aleksiev B. (2008). An evaluation of the implementation of the Cramer classification scheme in the Toxtree software. SAR QSAR Environ. Res..

[34] Matthews C., Moran F., Jaiswal A.K. (2021). A review on European Union’s strategy for plastics in a circular economy and its impact on food safety. J. Clean. Prod..

[35] Plastic Recyclers Europe (PRE) Flexible Polyethylene Recycling in Europe: Accelerating the Transition towards Circular Economy. https://www.pac.gr/bcm/uploads/flexible-pe-recycling-in-europe_june-2019.pdf.

[36] Commission Regulation (EC) No 282/2008 of 27 March 2008 on Recycled Plastic Materials and Articles Intended to Come into Contact with Foods and Amending Regulation (EC) No 2003/2006 (and its Successive Amendments). http://data.europa.eu/eli/reg/2008/282/2015-10-26.

[37] Fullana Font A., Lozano Morzillo A. (28 october 2013). Method for Removing Ink Printed on Plastic Films.

[38] (2021). Compound Discoverer 3.2 Software. https://www.thermofisher.com/es/es/home/industrial/mass-spectrometry/liquid-chromatography-mass-spectrometry-lc-ms/lc-ms-software/multi-omics-data-analysis/compound-discoverer-software.html.

[39] Food Contact Additives (FCA) (2020). Risk Assessment of Non-Listed Substances (NLS) and Non-Intentionally Added Substances (NIAS) under the Requirements of Article 3 of the Framework Regulation (EC) 1935/2004, Version 3.0. https://fca.cefic.org/wp-content/uploads/2021/02/FCA_Risk_Assessment_Guidelines_v30-1.pdf.

